# Temperature-Dependent Microwave Responses of Polyethylene Pipe Butt-Fusion Joints

**DOI:** 10.3390/polym18212734

**Published:** 2026-11-03

**Authors:** Jiao Shi, Gongtian Shen, Kexue Jing

**Affiliations:** 1 School of Quality and Technical Supervision, Hebei University, Baoding 071000, China; 20249020005@stumail.hbu.edu.cn; 2 China Special Equipment Inspection and Research Institute, Beijing 100029, China; 3 Hebei Special Equipment Supervision and Inspection Research Institute, Shijiazhuang 050061, China

**Keywords:** polyethylene pipe, butt fusion, welding temperature, microwave nondestructive testing, cold fusion, scanning electron microscopy, tensile properties

## Abstract

Welding temperature affects the structure and performance of polyethylene (PE) butt-fusion joints. PE100 DN200 SDR11 joints were prepared at six temperatures (150–300 °C), with pressure, heating time, and changeover time held constant. Microwave line-scan signals over 6–14 GHz were acquired, and the normalized weld-to-background amplitude contrast (NWBCA), the complex spectral difference root mean square (CSD-RMS), and the background-corrected CSD-RMS (BG-CSD-RMS) were calculated. Macroscopic bead measurements, scanning electron microscopy, and tensile fracture observations provided complementary evidence. Relative to the 230 °C reference, both complex spectral metrics increased toward the low- and high-temperature conditions. NWBCA rose from 0.037 at 150 °C to 0.143 at 230 °C and fell to 0.094 at 300 °C. The 150 °C specimens fractured in a brittle manner in the weld, whereas the 300 °C specimens fractured in a ductile manner in the heat-affected region. Similar spectral deviations therefore accompanied different fracture behaviors. Overall, the three metrics described temperature-dependent joint responses within this specimen series and could serve as a basis for screening deviations from the nominal welding-temperature window.

## 1. Introduction

Polyethylene (PE) pipes combine corrosion resistance, low weight, flexibility, efficient installation, chemical resistance, and strong resistance to slow crack growth, and are therefore widely used in municipal gas, water and wastewater, petrochemical, and selected auxiliary systems in nuclear power plants [1]. Chinese standards for buried PE gas-pipeline systems define the materials, dimensions, and application limits of PE pipes and piping systems [2,3], while a recent review emphasized that the long-term reliability of PE pipelines is jointly controlled by material properties, joint quality, environmental exposure, and defect evolution [1]. For medium- and large-diameter PE pipes, butt fusion is among the most common joining methods. The technical rules for PE gas-pipe welding [4] and the butt-fusion tensile-test standard [5] provide complementary requirements for joint preparation and testing. The fusion zone is consequently both the region at which structural continuity is established and a region sensitive to in-service failure.

A PE butt-fusion joint is formed by heating the pipe ends to produce molten layers, pressing the ends together, and allowing the assembly to cool under controlled conditions. Joint quality depends on welding temperature, pressure, heating time, and changeover time. Of these parameters, welding temperature directly controls molten-layer thickness, melt viscosity, segmental mobility, and interdiffusion across the interface, and is therefore critical for distinguishing low-temperature cold fusion, adequate fusion, and overheating. Studies of thermoplastic interfacial fusion kinetics show that joint formation is governed by chain interdiffusion and entanglement: recovery of interfacial stiffness is associated with segmental diffusion, whereas interfacial strength depends more strongly on chain entanglement [6]. Insufficient temperature limits melting at the pipe ends, reduces the population of chains able to cross the interface, and promotes weak bonding. Excessive temperature, in contrast, may cause excessive melt flow, enlarge the heat-affected zone (HAZ), and increase local molecular orientation. Li et al. showed that welding modifies crack-growth behavior and fracture morphology in PE pipes, confirming that microstructural evolution in the joint affects long-term reliability [7]. Departures from the temperature window may thus cause degradation at both extremes: insufficient fusion at low temperature and overheating-induced or HAZ anomalies at high temperature.

Current methods for evaluating PE joint quality include destructive tests, such as tensile testing, and nondestructive methods based on ultrasonics, radiography, and infrared thermography. Ultrasonic testing can identify voids, inclusions, and lack of fusion that produce marked acoustic-impedance discontinuities. Its sensitivity to cold-fusion or overheating states expressed mainly through inadequate chain interdiffusion or subtle microstructural differences is, however, limited by strong acoustic attenuation in PE and sensitivity to coupling on curved surfaces. Radiography is sensitive to volumetric density differences but provides limited contrast for weak interfaces and small dielectric or microstructural variations, in addition to requiring radiation protection. Infrared methods can reveal abnormal process or surface-temperature fields, but provide limited direct information on the internal condition of a cooled joint [8].

PE is a semicrystalline dielectric with low electrical conductivity and pronounced viscoelasticity. Cold fusion and overheating may therefore manifest primarily as changes in interfacial continuity, local compactness, crystalline or oriented structures, and microcracking. Microwave methods exploit the interaction of electromagnetic waves with dielectric media and are sensitive to amplitude and phase changes caused by variations in permittivity, dielectric loss, and interfacial continuity. They are also non-contact, require no couplant, and are suitable for rapid scanning. Studies published in NDT & E International have shown that K- and W-band frequency sweeps can combine reflection-coefficient amplitude and phase to detect and size defects beneath coatings [9]; near-field microwave characterization can identify and quantitatively evaluate phase-change inclusions in honeycomb composites [10], and non-contact near-field cavity-perturbation measurements can quantitatively characterize dielectric state [11]. These findings indicate that multifrequency or wideband measurements provide richer spectral information than single-frequency measurements and support their application to PE butt-fusion joints.

Previous studies have established that microwave signals respond to structural anomalies in PE pipes and butt-fusion joints. Schmidt et al. and Stakenborghs used microwave interference scanning and volumetric inspection, respectively, to detect defects in nonmetallic pipes and cold-fusion joints in high-density polyethylene (HDPE) pipes [12,13]. Zhu et al. found that cracks and cold-fusion defects alter microwave scan images and used tensile and true-density tests to confirm associated changes in compactness and mechanical performance [14,15]. Zhao linked weld defects to changes in the amplitude and phase of reflected signals and compared field-joint results with digital radiography [16]. Niu et al. further related the microwave response of cold-fusion joints to mechanical properties, fracture morphology, and molecular-chain structure [17,18]. Near-field imaging and line-scan studies have also shown that clutter suppression or hierarchical recognition can enhance and quantify the spatial response of internal defects in PE pipes [19,20]. Using focused antennas and resonant cavities, Wang et al. observed changes in resonance response, microstructure, density, and crystallinity in HDPE joints fabricated at different temperatures [21], while Pan et al. showed that resonant parameters respond to cracks, voids, and inclusions [22].

Despite this progress, three limitations remain. First, most specimen sets compare only cold-fusion and reference joints or a few discrete temperatures; continuous coverage from insufficient low-temperature melting through adequate fusion to high-temperature overheating is lacking, which obscures the overall response between 150 and 300 °C. Second, substantial differences among interference scanning, volumetric inspection, resonant cavities, and resonant probes limit comparability. Some reports provide insufficient details on the operating band, frequency increment, or full-band processing, and many rely on a single frequency, narrowband resonance parameters, or pseudocolor images. Third, existing studies mainly address whether a defect can be detected, rather than how amplitude, phase, and complex spectral features evolve with welding temperature or whether low-temperature cold fusion and high-temperature overheating produce distinct microwave signatures. A continuous temperature series measured under a consistent instrument configuration, coupled with wideband complex-signal analysis and macroscopic, microstructural, and mechanical validation, is therefore needed.

To address these limitations, the present study uses a 6–14 GHz wideband scan rather than a single narrowband frequency. Weld geometry, interfacial continuity, compactness, and microstructure may affect different frequencies differently, whereas a single frequency is susceptible to local resonance, lift-off variation, and random noise and cannot fully describe the dielectric state of the joint. Preliminary frequency-sweep tests further showed that PE100 and its butt-fusion joints provide stable, measurable reflection responses across 6–14 GHz, with distinguishable amplitude and phase responses between the weld and base material. Integrating 201 frequency points across this band incorporates frequency-dependent information while reducing the influence of isolated points. Accordingly, six temperatures from 150 to 300 °C were selected to span insufficient low-temperature fusion, adequate fusion, and high-temperature overheating or HAZ anomalies. NWBC_A_, CSD-RMS, and BG-CSD-RMS were calculated from the wideband complex signals and related to weld geometry, SEM observations, and tensile parameters from type-A specimens to determine the temperature-dependent microwave response and its physical origin. In summary, this study contributes (i) a continuous six-temperature series spanning insufficient low-temperature fusion, adequate fusion, and high-temperature overheating, prepared under a single-factor design and measured with one consistent instrument configuration; (ii) a wideband 6–14 GHz complex-signal analysis with three complementary metrics that separately describe amplitude contrast, spectral deviation from the reference joint, and background-corrected local anomaly; and (iii) a multi-level validation linking the microwave signatures to directly measured bead geometry, SEM morphology, and tensile fracture behavior. To the best of our knowledge, this combination of a continuous welding-temperature series, wideband complex-signal metrics, and independent mechanical and microstructural validation has not been reported previously for PE butt-fusion joints.

## 2. Materials and Methods

### 2.1. Experimental Design and Workflow

**Figure 1:**
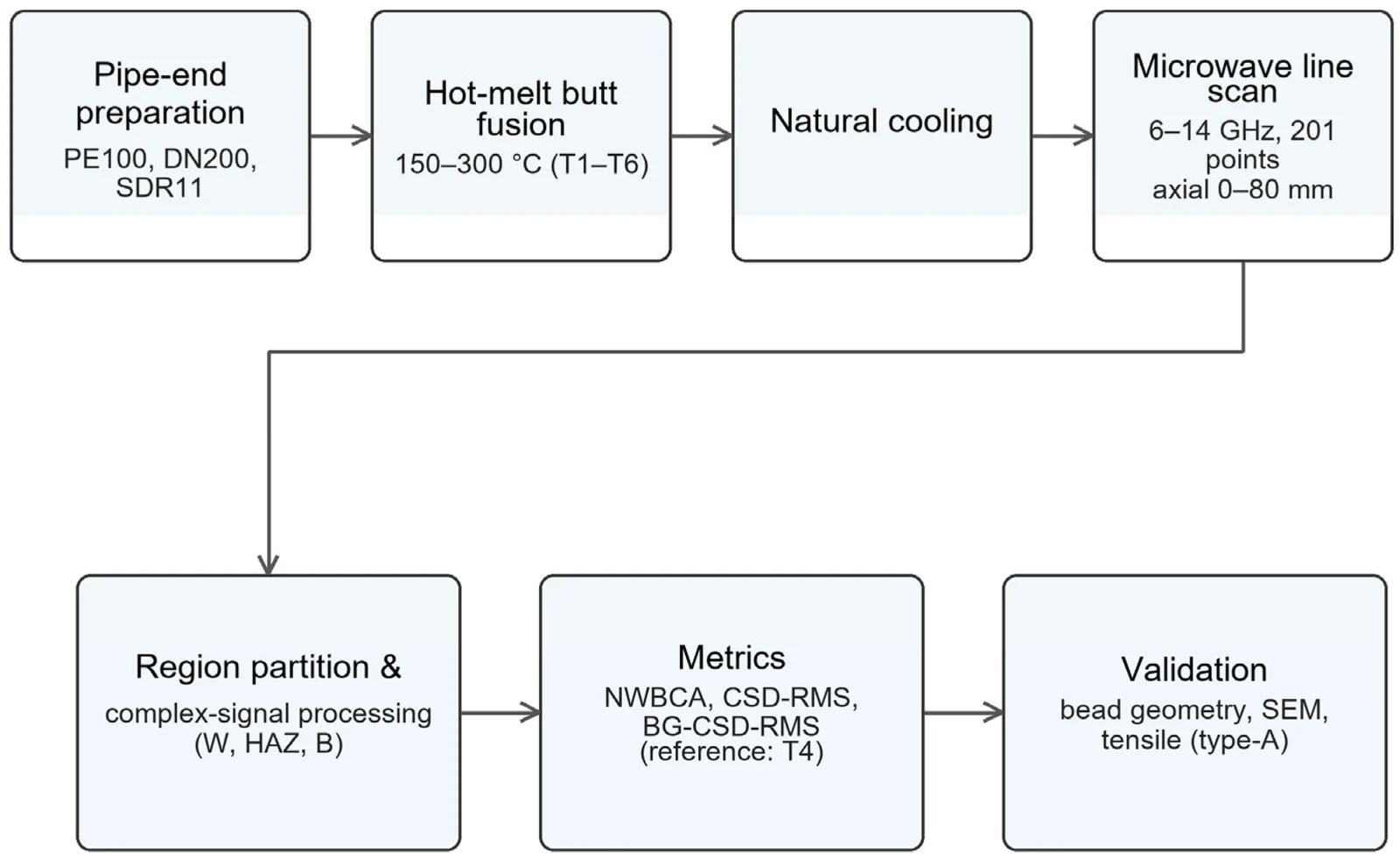
Overall workflow for characterizing and interpreting microwave signatures of PE pipe welds produced at different temperatures.

A single-factor design was used in which welding pressure, heating time, and changeover time were held constant while welding temperature was varied. The resulting specimen series covered insufficient low-temperature melting, adequate fusion, and high-temperature overheating or HAZ anomalies. Microwave line scanning, macroscopic geometric measurements, SEM observations, and tensile validation using type-A specimens were then performed sequentially. Rather than analyzing a single frequency, the analysis quantified differences in the full-band complex response between the weld and base-material background regions, thereby establishing a closed-loop interpretation linking macroscopic geometry, microstructure, microwave response, and mechanical behavior. The workflow is shown in Figure 1.

### 2.2. Specimen Preparation and Welding Temperature Setting

The test object was PE100 pipe (nominal diameter DN200, standard dimension ratio SDR11), and the joints were prepared by hot-melt butt fusion using an automatic butt-fusion welding machine (Yada PBF 315, Baoding, China). The pipe was a commercially available PE100 grade (BorSafe™ HE3490-LS, Borouge) with a bimodal high-density molecular structure; according to the supplier’s product data sheet, the compound density is 960 kg/m^3^ (ISO 1183) and the melt flow rate is 0.25 g/10 min (190 °C/5 kg, ISO 1133). The pipes were manufactured by pipe extrusion; numerical molecular-weight data are not specified in the supplier’s data sheet. To study the single-factor influence of welding temperature, six temperature levels, T1–T6 (150, 180, 210, 230, 260, and 300 °C), were selected. T4 (230 °C) served as the nominal reference welding temperature, and all other process parameters were held constant: the welding pressure (machine gauge pressure) was 2.64 MPa, heat absorption time was 182 s, and the changeover time was 8 s. One joint was prepared for each temperature level, and three Type-A tensile specimens were subsequently machined from each joint in accordance with GB/T 19810—2005. The parameter design covers three typical regimes—insufficient low-temperature melting, nominal welding, and high-temperature over-welding/heat-affected-zone anomalies—as shown in Table 1.

**Figure 2:**
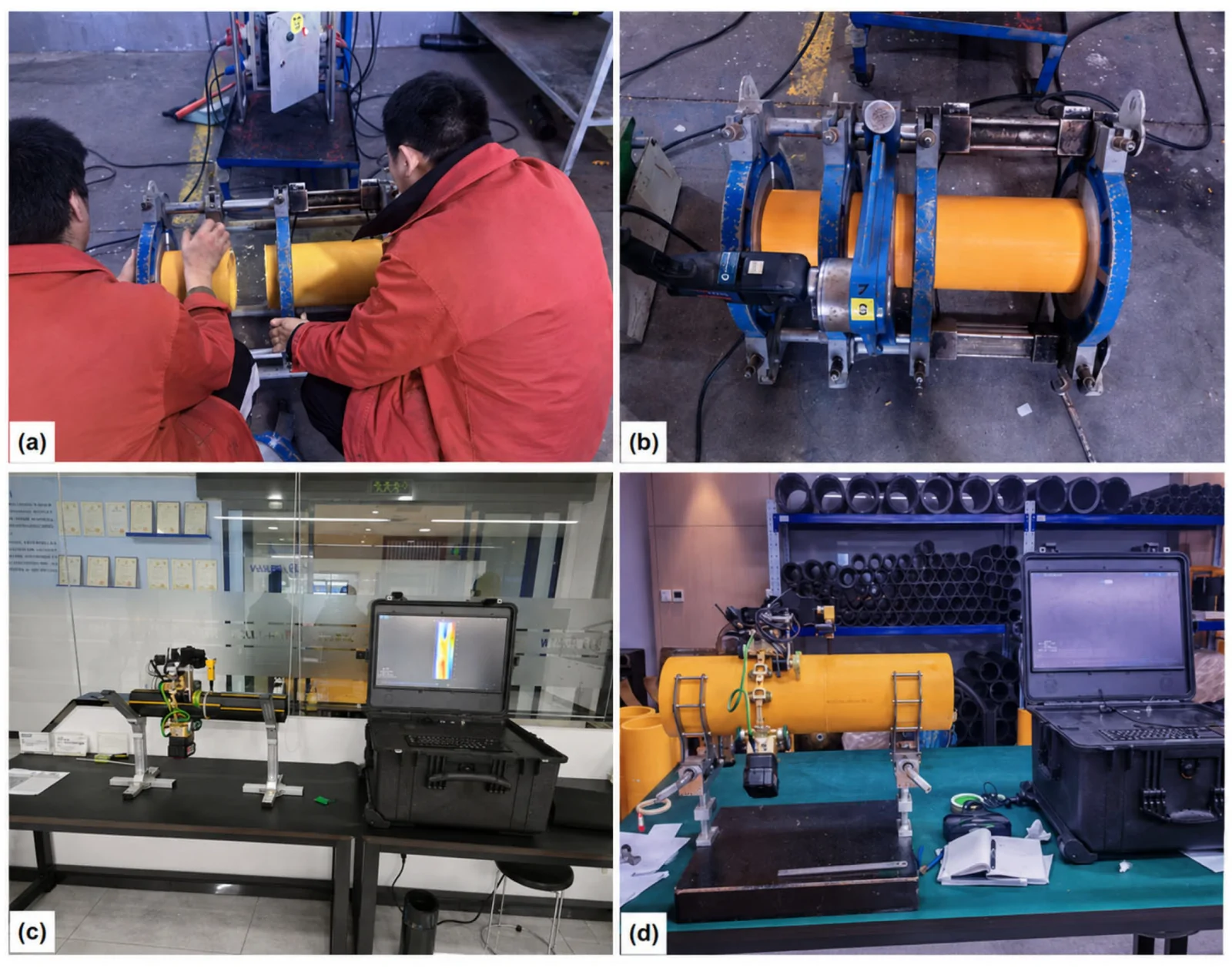
Specimen preparation and microwave detection process: (**a**) Specimen preparation by hot-melt butt welding; (**b**) joint clamping and welding process; (**c**) microwave detection system; (**d**) linear scanning detection process.

The hot-melt welding, microwave inspection system, and line-scan process are illustrated in Figure 2. To ensure comparability in the study of temperature variations, all specimens were prepared under identical conditions—namely, the same pipe specification, clamping method, pressure, heat absorption time, and switching time—and were then subjected to natural cooling followed by subsequent inspection.

### 2.3. Microwave Testing Instruments and Scanning Methods

Microwave measurements were obtained using a TS2010 multifrequency microwave PE-pipe inspection system (Henan Delang Intelligent Technology Co., Ltd., China). The system comprises a microwave source, a near-field probe, a control unit, and axial and circumferential scanning mechanisms, and operates in near-field reflection mode. The source covers 100 MHz–20 GHz with a maximum output power of 20 dBm. The probe used in this study has an effective response from 6 to 14 GHz, a voltage standing-wave ratio no greater than 1.4, a gain of 6–11 dB, and an approximate beam diameter of 7–8 mm at a lift-off distance of 3 mm. The axial stage has a travel range of 0–100 mm and nominal speed and increment of 1 mm/s and 1 mm, respectively.

During the experiments, lift-off was fixed at 3 mm and an axial line scan was conducted from 0 to 80 mm using a spatial increment of 5 mm. The frequency range was 6–14 GHz with a 0.04 GHz increment, yielding 201 frequency points at each spatial position. Probe orientation, lift-off, scanning direction, and instrument gain were held constant. At every spatial and frequency point, the system recorded the complex reflection signal, including X and Y coordinates, frequency, real and imaginary components, and frequency-point index. At each axial position, measurements were repeated at four circumferential positions spaced at 90° intervals, denoted by the clock-face orientations 12, 3, 6, and 9 o’clock; Y in the raw response maps therefore represents this discrete circumferential sampling index expressed as a clock-face orientation.

**Figure 3:**
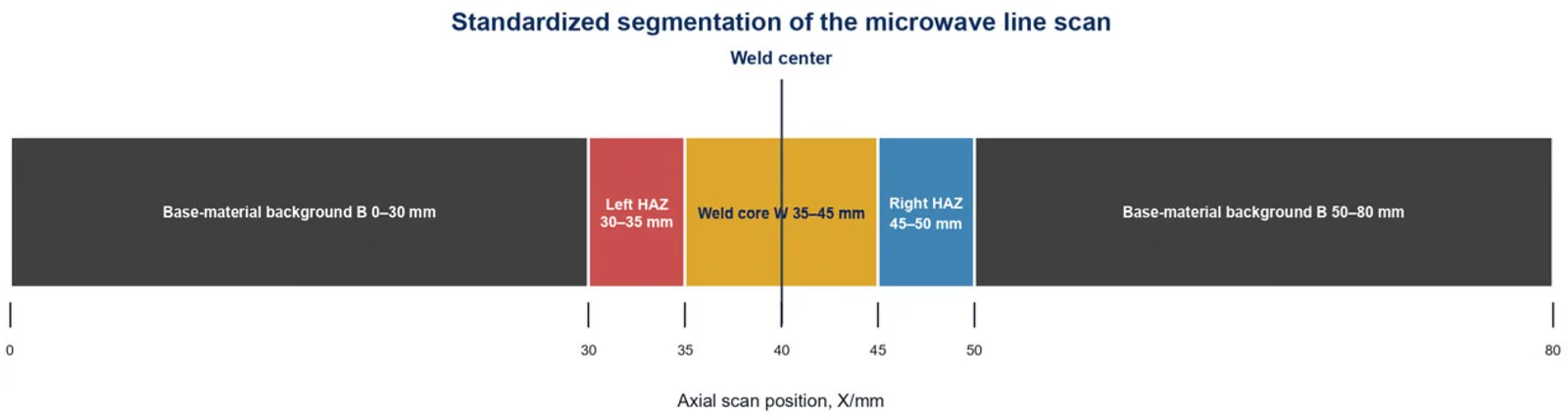
Standardized partition of the microwave line scan.

To reduce the effects of overall pipe curvature, local material variation, and one-sided background drift, regions were defined relative to the weld center (Figure 3 and Table 2). The interval X = 35–45 mm was designated as the weld core region W; X = 30–35 mm and X = 45–50 mm were treated as the two HAZ transition regions; and X = 0–30 mm together with X = 50–80 mm formed the base-material background region B. Given the 7–8 mm beam diameter at 3 mm lift-off, a 10 mm weld-core width was selected to cover the central weld response while accommodating center-location uncertainty and spatially discrete sampling, with minimal contamination from adjacent transition regions. Averaging the two base-material regions reduced bias arising from one-sided material variation and curvature.

### 2.4. Macroscopic Examination, SEM, and Tensile Validation

To elucidate the mechanism underlying the variation in microwave characteristic signals with welding temperature, this study simultaneously conducted macroscopic morphology analysis, scanning electron microscopy (SEM) microstructural characterization, and mechanical tensile testing. The macroscopic morphology analysis primarily documented the appearance of the weld seam’s edge/teeth lip, weld seam width, bead height, misalignment amount, longitudinal cross-sectional morphology, and the fracture position after tensile failure, providing an intuitive basis for assessing the formation of the molten layer and the state of melt extrusion. The weld seam width and bead height were measured using a vernier caliper; for each specimen, representative measurement points were selected for recording, and these measurements were then compared and analyzed in relation to the microwave anomalies and mechanical test results.

The SEM specimens were obtained from the central region of the weld joint, with a sampling size of approximately 8 mm × 8 mm × 10 mm. After cutting, surface cleaning, drying, and gold sputtering for conductive coating, the specimens were examined using a ZEISS GeminiSEM 560 (Carl Zeiss Microscopy Ltd., Cambridge, UK) field-emission scanning electron microscope. This study focuses on comparing the cross-sectional morphologies of the T1-T6 temperature-group specimens at a magnification of 10,000×, with an emphasis on characteristics such as interface continuity, microcracks, micropores, micro-fibrillar tearing, layered/plate-like structures, and local orientation, in order to elucidate the microscopic origin of the anomalous microwave response.

Mechanical tensile tests were conducted in accordance with GB/T 19810—2005 (identical to ISO 13953:2001) [5]. Three Type-A specimens were cut in parallel from each joint along the circumferential direction; the specimens were then trimmed to the required length and width, and the holes were drilled according to the Type-A template. The specimens were conditioned at 23 ± 2 °C for at least 6 h before testing. The thickness and the width (the distance between the two holes) of each specimen were measured twice and averaged. The tests were performed on a CMT4304 electronic universal testing machine (Shenzhen XinSanSi Material Testing Co., Ltd., Shenzhen, China) at a crosshead speed of 5 mm/min at 23 ± 2 °C until complete failure, and the relevant data were recorded. Maximum force, tensile strength, fracture location, and failure appearance were recorded for each specimen, and the values reported in Table 5 are the means of the three specimens per joint. Fracture location and appearance were used as complementary observations of joint behavior rather than as stand-alone proof of the underlying microscopic mechanism.

## 3. Microwave Signal Processing

Complex signals were converted to amplitude and phase, averaged within predefined regions, and compared with the 230 °C reference joint as described below.

### 3.1. Complex Signal and Amplitude-Phase Conversion

At the i-th spatial scanning point and the k-th frequency point, the complex signal output by the microwave detection system can be expressed as:

(1) *Z_i_*(*f_k_*) = *Re_i_*(*f_k_*) + *jIm_i_*(*f_k_*)

where *Z_i_*(*f_k_*) is the complex microwave signal; and *Re_i_*(*f_k_*) are its real and imaginary components, respectively; *Im_i_*(*f_k_*) is the imaginary unit, with; and *j* denotes the j-th frequency point. Here, *j*^2^ = −1*f_k_kk* = 1, 2, ⋯ , *KK* = 201.

The amplitude and phase are obtained from the complex signal as follows:

(2) $$A_{i}(f_{k})=\sqrt{Re_{i}(f_{k}{)}^{2}+Im_{i}(f_{k}{)}^{2}}$$

(3) *φ_i_*(*f_k_*) = atan2[*Im_i_*(*f_k_*), *Re_i_*(*f_k_*)]

When necessary, the amplitude can also be converted to dB:

(4) *A_dB_*_,_*_i_*(*f_k_*) = 20*log*_10_*A_i_*(*f_k_*)

Amplitude describes the magnitude of the measured reflection signal, whereas phase describes its angle relative to the instrument reference; Equations (1)–(4) follow the standard definitions of the complex reflection signal and its amplitude–phase conversion [23]. Wavelet-packet-based signal processing and millimeter-wave near-field microscopy and phase imaging [24,25] have likewise been reported to enhance microwave NDT sensitivity to subtle dielectric and structural anomalies. The following metrics retain either amplitude or the complete complex signal.

### 3.2. Regional Averages for the Weld and Parent-Material Background

Based on the regional definitions in Section 2.3, X = 35–45 mm is defined as the weld core area W, and the two sides of X = 0–30 mm and X = 50–80 mm are merged and defined as the base metal background area B. At each frequency point *f_k_*, the average amplitude and average complex signal of the weld zone and the base metal background zone are calculated, respectively.

The mean amplitude in the weld region is:

(5) $${\bar{A}}_{W}(f_{k})=\frac{1}{N_{W}}\sum_{i\in W}A_{i}(f_{k})$$

and that in the parent-material background is:

(6) $${\bar{A}}_{B}(f_{k})=\frac{1}{N_{B}}\sum_{i\in B}A_{i}(f_{k})$$

The mean complex signal in the weld region is:

(7) $${\bar{Z}}_{W}(f_{k})=\frac{1}{N_{W}}\sum_{i\in W}Z_{i}(f_{k})$$

and that in the parent-material background is:

(8) $${\bar{Z}}_{B}(f_{k})=\frac{1}{N_{B}}\sum_{i\in B}Z_{i}(f_{k})$$

In these formulae, *N_W_* and *N_B_* represent the number of effective scanning points within the weld zone and the base material background zone, respectively. By combining the base material regions on both the left and right sides into a single background zone, the impact of unilateral material fluctuations, pipeline curvature, and local scanning errors on the background estimation can be reduced, thereby making the response differences between the weld zone and the background zone more comparable.

### 3.3. Normalized Weld-to-Background Amplitude Contrast (NWBC_A_)

To characterize the amplitude difference between the weld zone and the base material background region, this study defines the normalized weld-to-background amplitude contrast (*NWBC_A_*). This indicator evaluates the degree of amplitude deviation between the weld zone and the base material zone across the entire frequency range; its calculation formula is as follows:

(9) $$\mathrm{NWB}C_{A}=\frac{\sum_{k=1}^{K}|{\bar{A}}_{W}(f_{k})-{\bar{A}}_{B}(f_{k})|}{\sum_{k=1}^{K}{\bar{A}}_{B}(f_{k})}$$

### 3.4. Complex Spectral Difference Root-Mean-Square Value (CSD-RMS)

To further leverage the complex information jointly contained within both the real and imaginary parts, this paper introduces the complex spectrum difference root-mean-square value (CSD-RMS). This metric uses the standard welded-temperature specimen T4 as a reference to evaluate the overall deviation of the average complex spectrum of the weld zone in the specimen under test relative to the standard weld condition:

(10) $$\mathrm{CSD}-\mathrm{RMS}=\sqrt{\frac{1}{K}\sum_{k=1}^{K}{|{\bar{Z}}_{W,s}\left(f_{k}\right)-{\bar{Z}}_{W,T4}\left(f_{k}\right)|}^{2}}$$

where ${\bar{Z}}_{W,s}(f_{k})$ is the average complex signal of the weld zone of the sample to be tested, and ${\bar{Z}}_{W,T4}(f_{k})$ is the average complex signal of the weld zone of the standard welding temperature sample T4.

Compared to the simple amplitude indicator, the CSD-RMS simultaneously retains both amplitude and phase information, enabling a more comprehensive reflection of the deviation of the microwave complex spectrum in the weld zone relative to that of a standard weld. When excessively low welding temperatures result in insufficient interface fusion, or when excessively high welding temperatures cause abnormal microstructure in the heat-affected zone, the complex spectrum in the weld zone may deviate from the standard reference spectrum, leading to an increase in this indicator.

### 3.5. Background-Corrected Complex Spectral Difference Root-Mean-Square Value (BG-CSD-RMS)

Specimen-level differences in the pipe and measurement setup may influence direct spectral comparisons. BG-CSD-RMS subtracts the parent-pipe spectrum of each specimen before comparison with the reference. Whether this correction reduces variation in practice remains to be tested with replicate specimens and scans.

For each specimen, the complex difference between the weld and parent-material background is first calculated as:

(11) $$\Delta Z_{s}(f_{k})={\bar{Z}}_{W,s}(f_{k})-{\bar{Z}}_{B,s}(f_{k})$$

For reference specimen T4, the corresponding weld-to-background difference is:

(12) $$\Delta Z_{T4}(f_{k})={\bar{Z}}_{W,T4}(f_{k})-{\bar{Z}}_{B,T4}(f_{k})$$

BG-CSD-RMS is then defined as:

(13) $$\mathrm{BG}-\mathrm{CSD}-\mathrm{RMS}=\sqrt{\frac{1}{K}\sum_{k=1}^{K}{|\Delta Z_{s}\left(f_{k}\right)-\Delta Z_{T4}\left(f_{k}\right)|}^{2}}$$

### 3.6. Interpretation of the Microwave Indicators

The three aforementioned indicators, respectively reflect the microwave response characteristics of the weld seam from different perspectives. NWBCA primarily reflects the amplitude difference between the weld seam region and the base material background region; CSD-RMS indicates the overall deviation of the complex spectrum of the weld seam region in the test specimen relative to that of the standard weld seam; whereas BG-CSD-RMS, after subtracting the base material background contribution from the test specimen, further quantifies the degree of abnormal local response of the weld seam relative to the standard weld seam. These three indicators do not represent a simple substitution relationship; rather, they form a progressive evaluation framework characterized by “amplitude difference–complex spectrum deviation–background-corrected local anomaly”. The symbols used in the preceding formulas and their physical meanings are summarized in Table 3.

## 4. Microwave Results

### 4.1. Multi-Frequency Line-Scan Results Versus Weld Zone Response Characteristics

**Figure 4:**
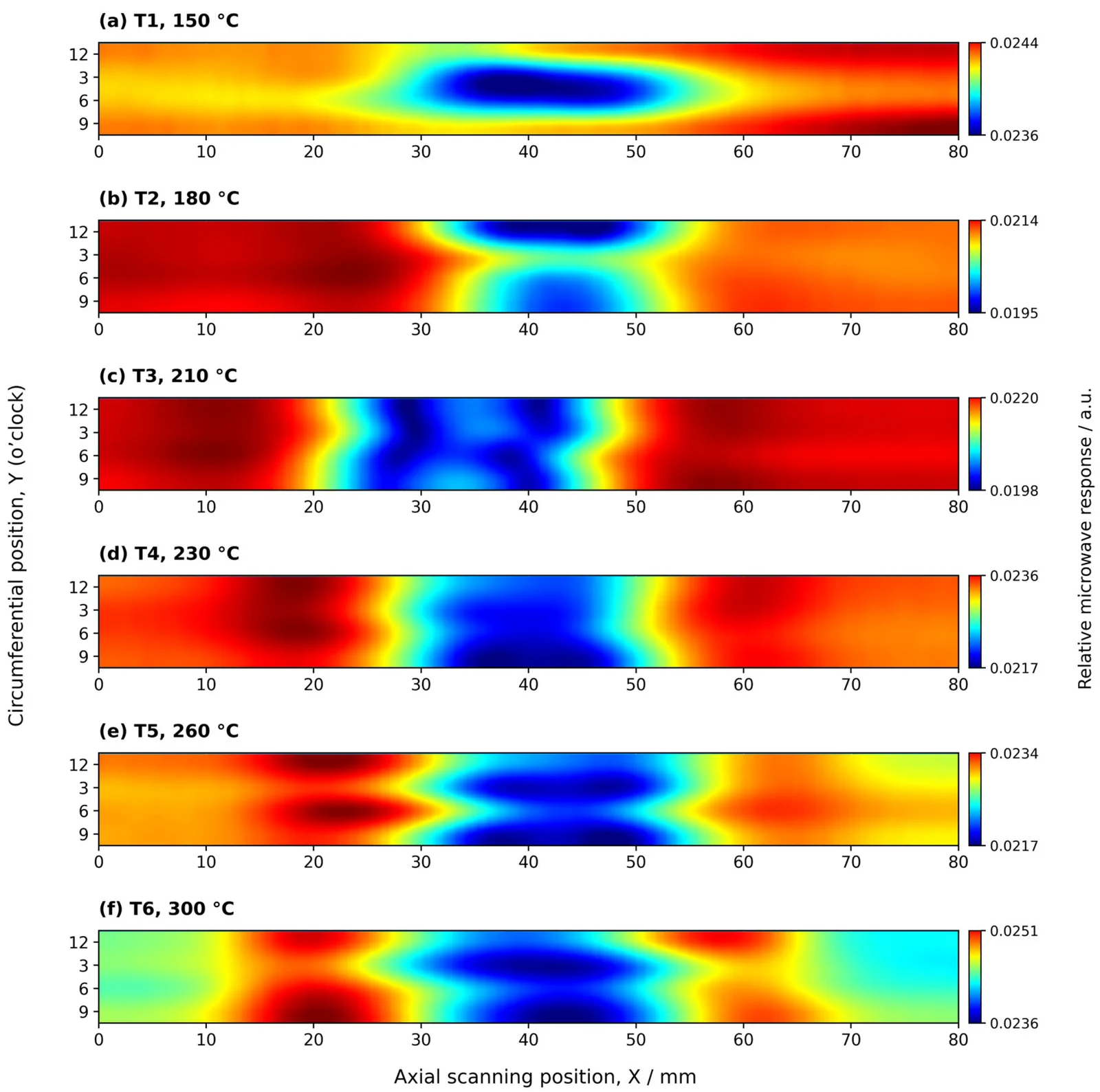
Raw microwave line-scan responses of specimens T1–T6.

Figure 4 compares representative microwave response maps for T1–T6 under the stated measurement settings. Position X runs along the pipe axis, while Y identifies circumferential sampling positions. The maps show the spatial extent and continuity of the joint response; apparent differences should be interpreted together with the frequency-domain metrics.

The central response varies among specimens in apparent width and boundary definition. Since geometry, probe footprint, and dielectric properties can all affect the reflected signal, these maps alone do not identify a particular microscopic defect.

Therefore, this study integrates the original complex-valued data from each spatial position across 201 frequency points to mitigate the influence of display grayscale levels and local fluctuations on the interpretation results. Following the methodology described in Section 3, the region defined as X = 35–45 mm is designated as the weld core region (W), while the base-material regions at both X = 0–30 mm and X = 50–80 mm are combined into the background region (B). The regional mean, full-frequency-band integral, and background correction are calculated separately for the weld region and the base-material background region. Table 4 presents the calculation results for three indicators—NWBC_A_, CSD-RMS, and BG-CSD-RMS—used to characterize the amplitude comparison between the weld region and the substrate region, the deviation of the weld complex spectrum relative to the reference sample (the 230 °C nominal reference joint, T4), and the local complex spectrum anomalies after subtracting the base-material background contribution from the sample; these results provide a quantitative basis for the subsequent analysis of temperature response patterns and underlying mechanisms.

### 4.2. Temperature Dependence of Microwave Indicators

**Figure 5:**
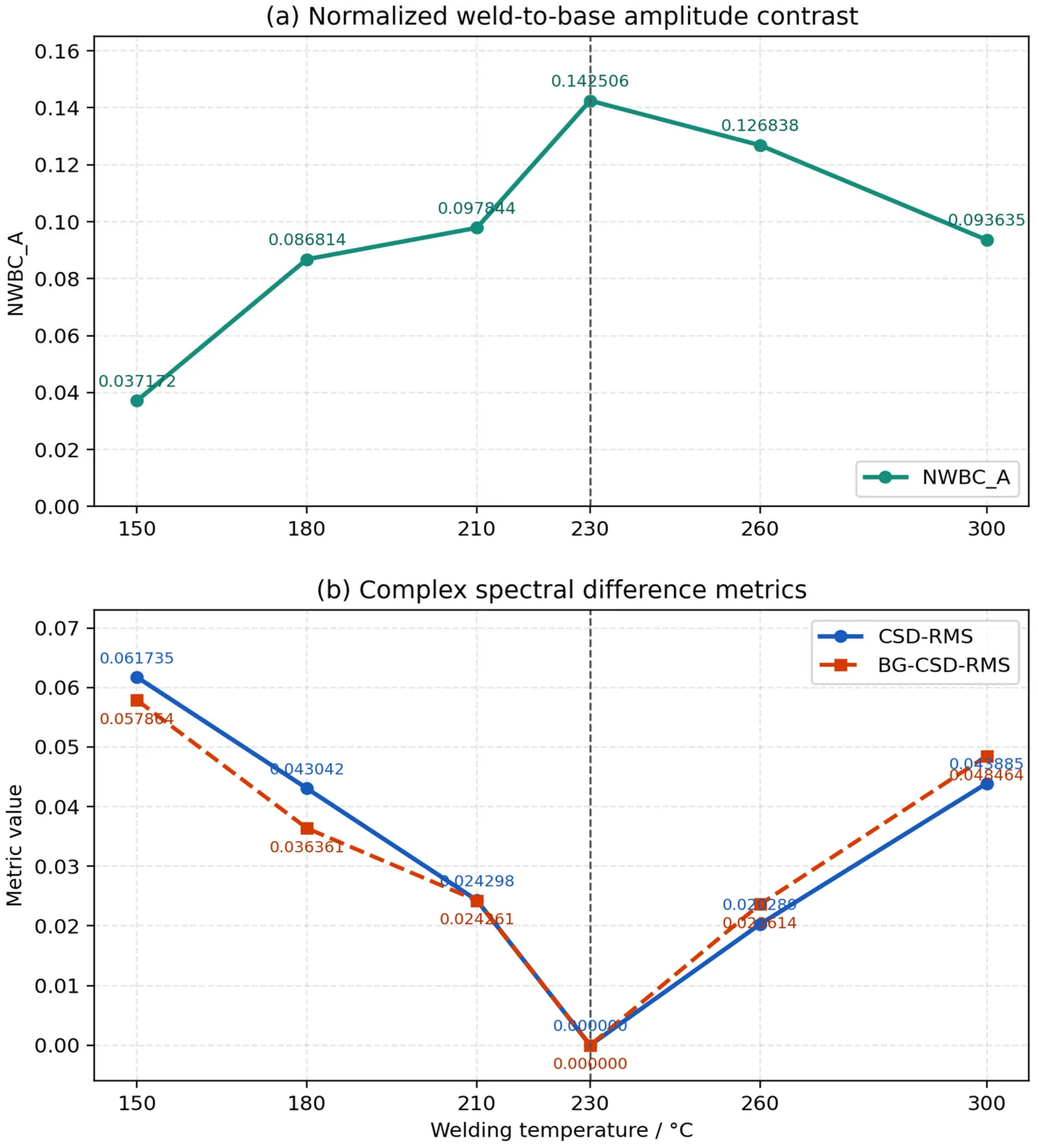
The curve of microwave quantification index changing with welding temperature: (**a**) NWBC_A_; and (**b**) CSD-RMS and BG-CSD-RMS.

To further quantify the line-scan response differences in the specimens at different temperatures in Figure 4, this study uses the T4 (230 °C) standard welding-temperature specimen as the reference, and calculates NWBC_A_, CSD-RMS, and BG-CSD-RMS for specimens T1–T6, respectively, as shown in Table 4. Figure 5 presents the trends of the three microwave evaluation indicators as functions of welding temperature in the form of line graphs.

NWBC_A_ first increased and then decreased. It rose from 0.037172 for T1 to 0.086814 for T2, 0.097844 for T3, and 0.142506 for T4, before decreasing to 0.126838 for T5 and 0.093635 for T6. Unlike the two complex spectral difference metrics, NWBC_A_ reached a maximum, rather than a minimum, at T4.

CSD-RMS exhibited a “first decrease-then increase” pattern with welding temperature. The values for T1–T4 were 0.061735, 0.043042, 0.024298, and 0, respectively; after T4, T5 and T6 rebounded to 0.020289 and 0.043885, respectively. On the low-temperature side, the metric decreased continuously as temperature increased; on the high-temperature side, it increased continuously as the temperature continued to rise.

BG-CSD-RMS followed essentially the same trend as CSD-RMS. The values for T1–T4 were 0.057864, 0.036361, 0.024261, and 0, respectively; T5 and T6 were 0.023614 and 0.048464, respectively. This metric likewise reached its lowest point at T4 and increased toward both the low-temperature and high-temperature ends.

Thus, NWBC_A_ was greatest at the reference temperature, whereas both reference-relative complex metrics were zero, by definition, at T4 and increased toward the temperature extremes (Figure 5). These six observations do not establish a calibrated decision threshold.

### 4.3. Factors Affecting the Microwave Response

NWBC_A_ compares the weld-region amplitude with the parent-pipe amplitude within each specimen. Its maximum at T4 may reflect changes in bead geometry, transition-region extent, and material response. The present measurements cannot separate these contributions; a larger NWBC_A_ must not be equated with greater defect severity.

CSD-RMS compares each weld spectrum with that of T4, whereas BG-CSD-RMS compares the corresponding weld-minus-parent-pipe spectra. Both equal zero at T4 by construction. Elevated values for T1 and T6 indicate departure from this particular reference, not a common defect type. Background correction is mathematically defined here, but claims of improved repeatability or resistance to curvature and lift-off require a dedicated perturbation experiment.

## 5. Joint Morphology, Tensile Behavior, and Discussion

This section examines how the temperature-dependent microwave response relates to bead geometry, SEM appearance, and tensile fracture. The data support associations among these measurements; a causal chain through dielectric properties and molecular structure remains a hypothesis because those properties were not measured directly.

### 5.1. Relationship Between Macroscopic Weld Morphology and Microwave Response

**Figure 6:**
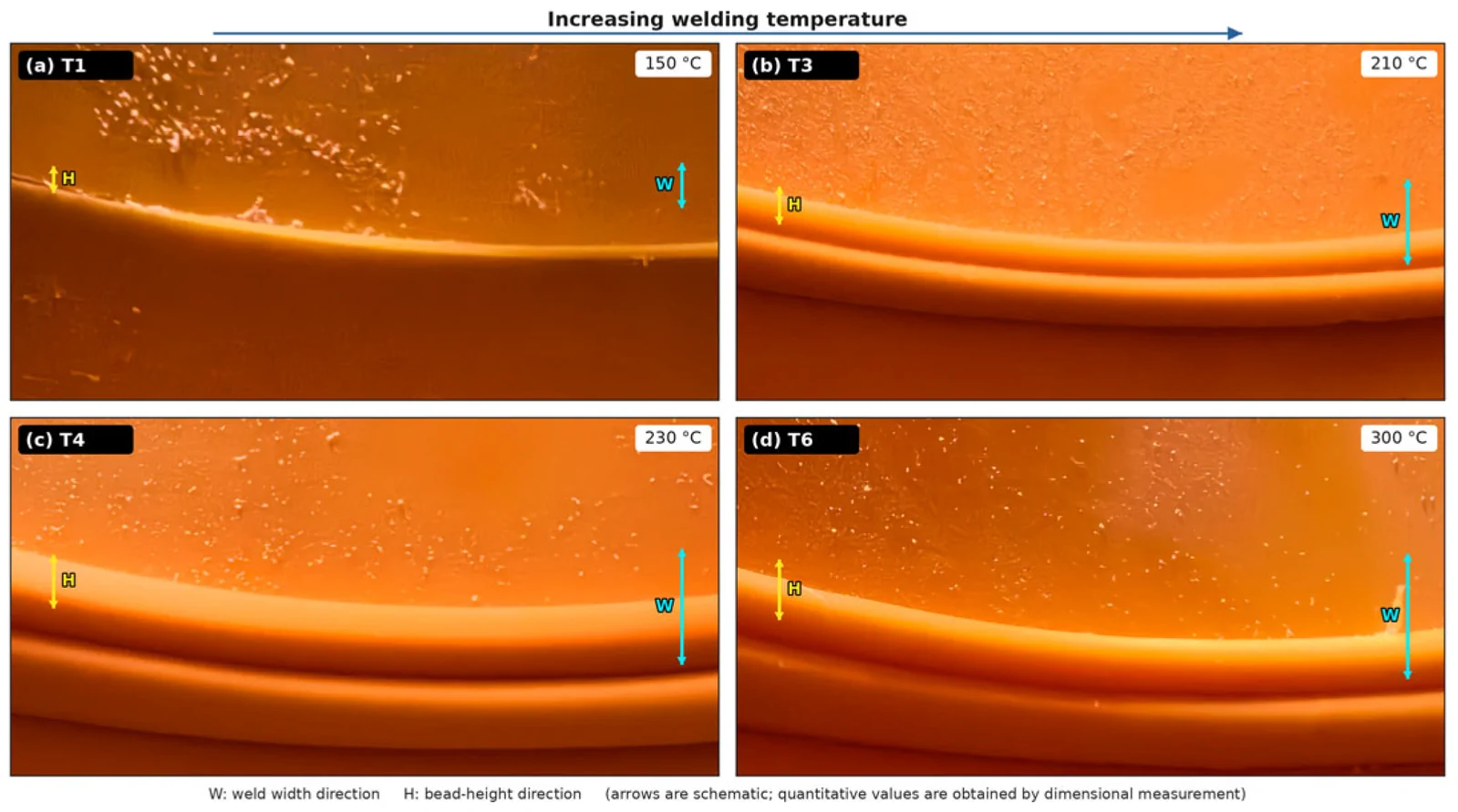
Macroscopic morphology of PE butt-fusion welds produced at different temperatures.

**Figure 7:**
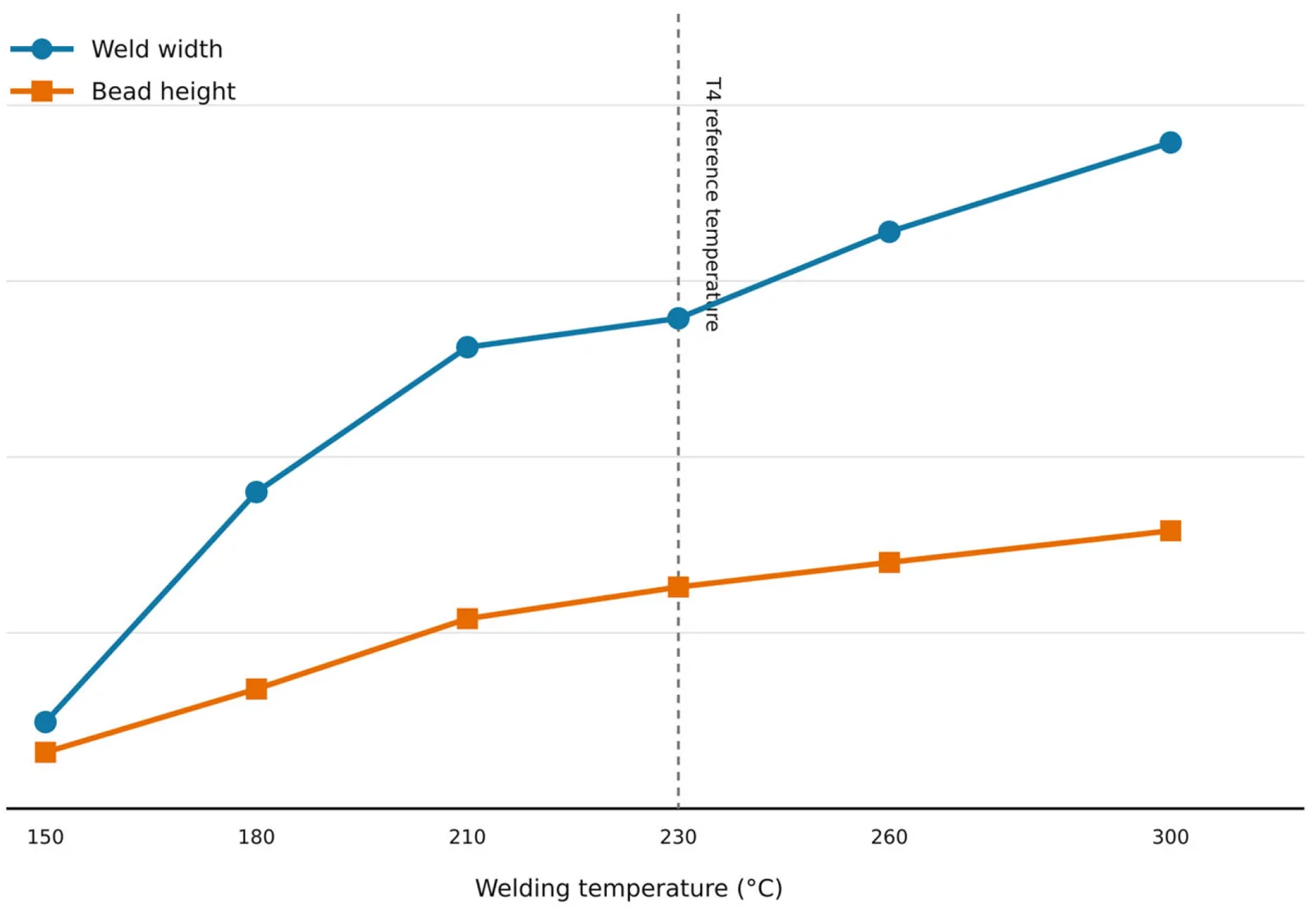
Effects of welding temperature on weld width and bead height.

The macroscopic appearance of the weld seam serves as an important visual indicator for determining whether the hot-melt butt welding process was performed adequately. As the welding temperature increases from 150 °C to 300 °C, the weld seam width and bead height generally exhibit an increasing trend, as shown in Figure 6 and Figure 7. Figure 6 illustrates the macroscopic morphology of PE hot-melt butt-welded seams at different welding temperatures: (a) T1, 150 °C; (b) T3, 210 °C; (c) T4, 230 °C; (d) T6, 300 °C. Here, W and H denote the weld seam width and bead height, respectively. At T1, the weld seam width is only 2.46 mm and the bead height 1.6 mm, consistent with severely inadequate molten-layer formation at the pipe ends and insufficient bead formation under the low-temperature condition. At T3, the weld seam width and bead height are 13.12 mm and 5.4 mm, respectively; at the T4 reference specimen, they are 13.94 mm and 6.3 mm, respectively, consistent with relatively stable weld-seam formation. At T6, the weld seam width and bead height reach 18.94 mm and 7.9 mm, respectively; the larger bead is consistent with enhanced melt flow at the high temperature, indicating a tendency toward over-welding or increased heat-affected-zone development.

This macroscopic formation variation is consistent with the variation pattern of NWBCA. At low temperatures (T1), the thin molten layer and insufficient bead formation reduce the amplitude difference between the weld-core region and the base-material background; consequently, NWBCA remains at a relatively low level. As the temperature rises to T3/T4, the molten layer becomes more fully developed and a stable geometric and material state is established in the weld zone, enhancing the amplitude contrast between the weld and the base material; as a result, NWBCA increases and reaches a higher level at T4. When the temperature further rises to T5/T6, the weld bead and the heat-affected zone expand further and the response boundary between the weld zone and the adjacent transition zones becomes relatively diffuse, leading to a decrease in the amplitude contrast between the weld and the base material; therefore, NWBCA declines at the high-temperature end.

T1 and T6 have very different bead dimensions but both deviate from the T4 complex spectrum. This observation suggests that bead geometry alone cannot account for the measured spectra. Contributions from geometry, local material properties, and probe coupling cannot be quantified separately using the present dataset.

To examine the relationship between bead geometry and the microwave amplitude contrast quantitatively, Pearson correlation coefficients were calculated between NWBCA and the measured weld width and bead height across the four temperatures for which bead dimensions were recorded (T1, T3, T4, and T6). The correlation coefficients were r = 0.71 for weld width and r = 0.75 for bead height. Because NWBCA varies nonmonotonically with temperature, these descriptive correlations (*n* = 4) indicate that bead geometry contributes to the amplitude contrast but does not uniquely determine it, consistent with the interpretation that the response boundary becomes more diffuse at the high-temperature end.

### 5.2. Relationship Between SEM Morphology and Microwave Response

PE is a semicrystalline polymer, and temperature-dependent fusion can alter melt flow and the morphology of a joint. SEM observations can describe fracture or prepared-section features, but they do not independently determine crystallinity, chain interdiffusion, dielectric permittivity, or microwave penetration. Specimens from the weld center (approximately 8 × 8 × 10 mm) were cut, cleaned, dried, gold coated, and examined with a ZEISS GeminiSEM 560 (Carl Zeiss, Oberkochen, Germany). The SEM observations are therefore interpreted together with the directly measured bead dimensions (Section 5.1) and the tensile fracture results (Section 5.3).

**Figure 8:**
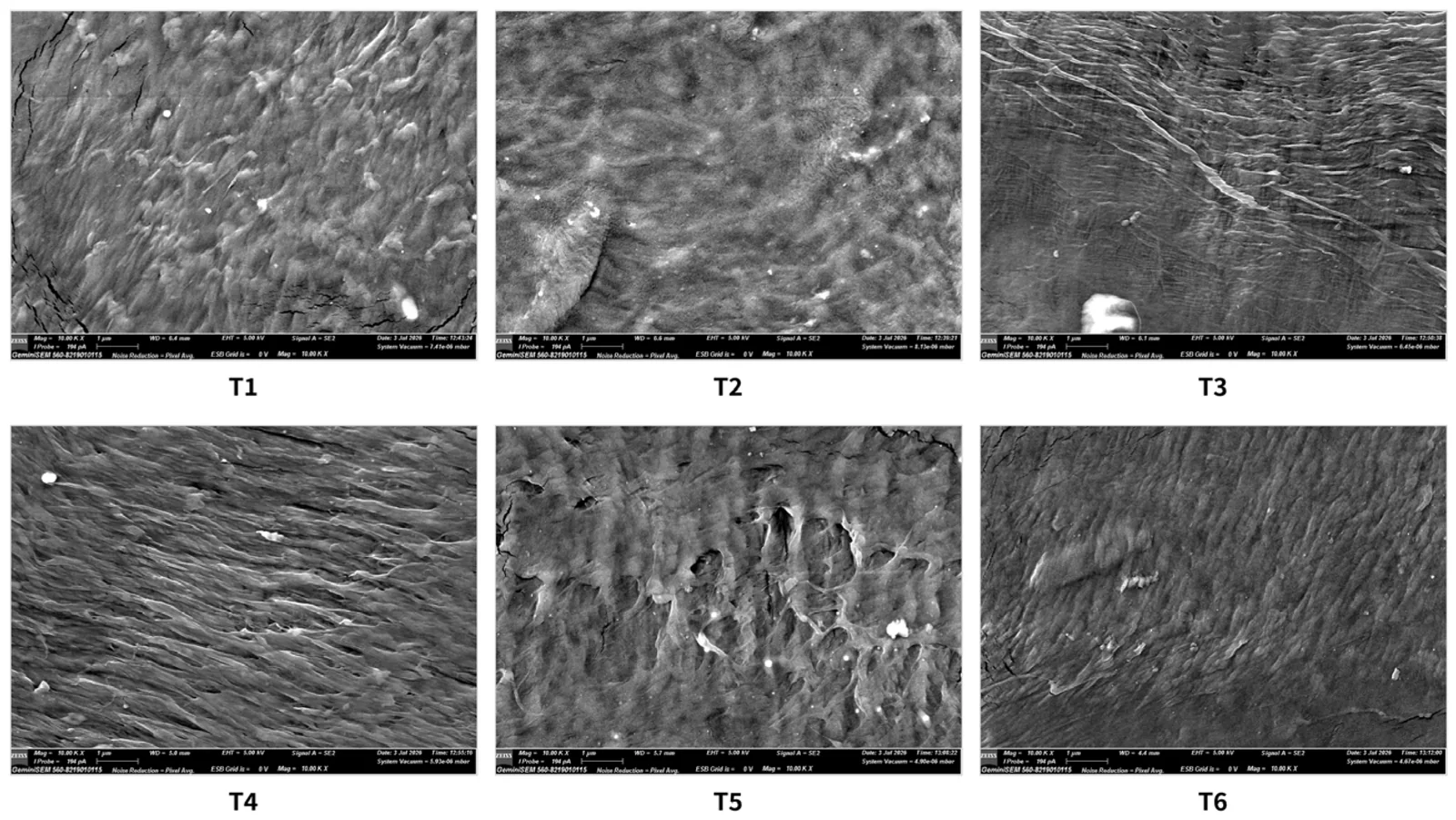
SEM fracture-section morphologies of specimens T1–T6 at 10,000× magnification.

Figure 8 compares SEM images of T1–T6 at 10,000×. Differences in surface continuity, fissures, and layered or fibrous appearance are described qualitatively. Without quantitative morphology measurements and confirmation of the imaged surface preparation and fracture history, these features should not be assigned uniquely to lamellae, molecular entanglements, or thermal degradation.

**Figure 9:**
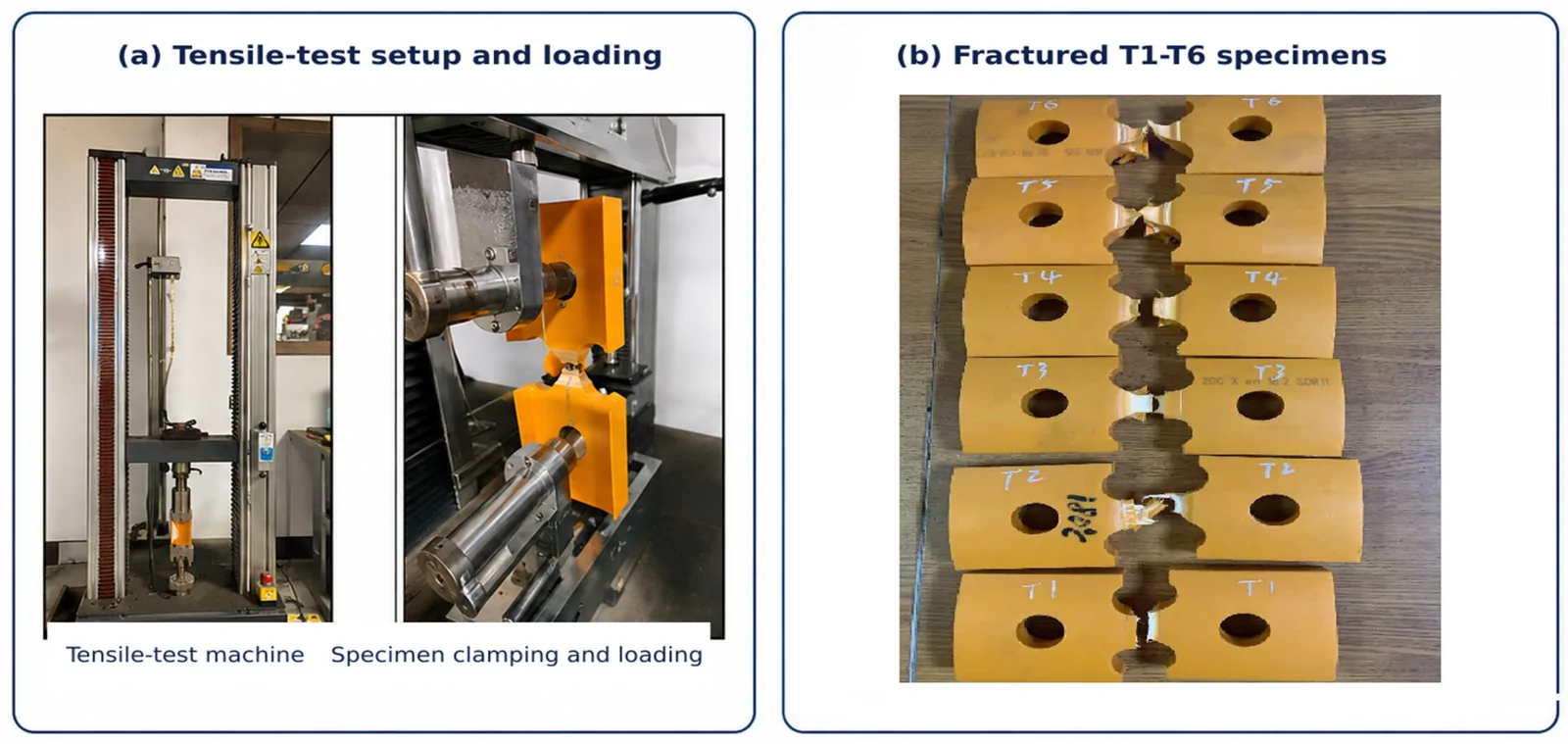
Type-A tensile testing and post-fracture appearances of the temperature-series specimens.

T1 shows localized discontinuities in the imaged region. Together with its small bead and brittle weld-region fracture, this is consistent with insufficient fusion at 150 °C. Its elevated complex spectral difference is also consistent with an altered joint response. The SEM image does not establish that reduced compactness or a particular dielectric change caused the microwave signal. The independent tensile results (Table 5, Figure 9) support this interpretation: the T1 joint fractured in a brittle manner at the weld with the lowest mean tensile strength (20.4 MPa) of the series.

The imaged surfaces of T2–T4 appear more continuous than T1 in the presented fields of view, while the reference-relative complex metrics decrease to zero at T4 by definition. These observations are compatible with improved fusion as temperature increases in this range, but cannot establish a quantitative relationship between SEM morphology and microwave response.

T5 and T6 display different surface textures from T1 in the presented SEM images. At T6 the larger bead, elevated spectral difference, and ductile fracture in the heat-affected region are compatible with a high-temperature or heat-affected response distinct from T1. The data do not establish chain orientation, polymer degradation, or the extent of the heat-affected zone. The independent tensile results (Table 5, Figure 9) are consistent with this observation: the T6 joint fractured in a ductile manner in the heat-affected region.

Accordingly, the two-sided spectral pattern is associated with different macroscopic and fracture observations at the two temperature extremes. Direct dielectric spectroscopy, crystallinity measurements, and repeated microstructural analyses are needed to test the proposed physical pathways.

### 5.3. Validation of Microwave Signals Using Mechanical Tensile Test Results

**Figure 10:**
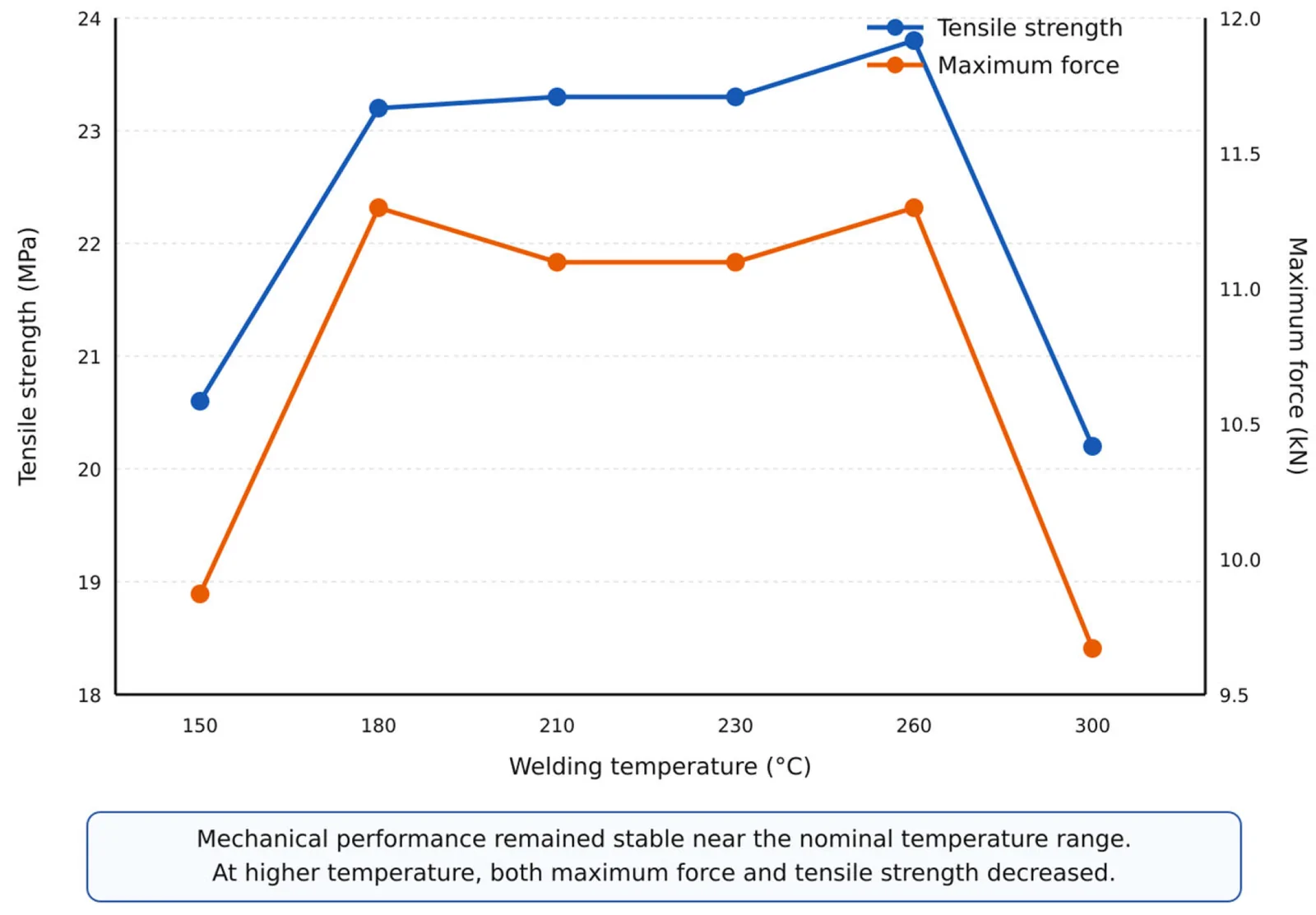
Temperature dependence of tensile strength and maximum force.

Tensile testing provided an independent comparison of joint performance and fracture location. Type-A specimens were tested with a CMT4304 universal testing machine in accordance with GB/T 19810—2005 [5]. The specimen dimensions and test speed follow the standard, and the number of specimens per joint (three) is detailed in Section 2.4. Maximum force, reported tensile strength, fracture location, and failure appearance are summarized in Table 5 and Figure 9 and Figure 10.

T1 reached a mean maximum force of 9.69 ± 0.19 kN and a mean tensile strength of 20.4 ± 0.2 MPa (n = 3) and fractured in the weld with a brittle appearance in all three specimens. This supports an interpretation of poor fusion under the 150 °C condition. Its large complex spectral difference is associated with this observation, but the present specimen series cannot establish a detection threshold or quantify predictive performance.

T2–T4 exhibited ductile fracture in the heat-affected region, with mean tensile strengths of 23.2 ± 0.1, 23.3 ± 0.1, and 23.4 ± 0.2 MPa, respectively (n = 3 per joint). Over this temperature range, the complex spectral metrics approached the T4 reference value. Replicate tests and uncertainty estimates are required to assess differences between neighboring temperatures.

T6 reached a mean maximum force of 9.68 ± 0.01 kN and a mean tensile strength of 22.9 ± 1.0 MPa (n = 3) and fractured in the heat-affected region with a ductile appearance in all three specimens. Its spectral deviation therefore accompanies a different fracture mode from T1. High-temperature melt flow or heat-affected changes are plausible explanations, but thermal degradation and molecular orientation were not directly demonstrated.

Together, the fracture observations show why a spectral distance from one reference joint cannot identify defect type on its own. T1 and T6 both differ from T4, but their bead dimensions and fracture modes differ. The suggested benefit of BG-CSD-RMS over CSD-RMS also remains unverified without replicate and nuisance-variable tests.

Quantitatively, the mean tensile strengths at 180–260 °C (T2–T5) form a plateau of 23.2–23.4 MPa, whereas the value at 150 °C (T1) is markedly lower (20.4 ± 0.2 MPa, approximately 12% below the plateau). At 300 °C (T6), the mean strength is 22.9 ± 1.0 MPa (n = 3), slightly below the plateau on average (approximately 2%), with the individual values ranging from 21.9 to 23.9 MPa; together with the largest bead and the elevated spectral difference, this indicates a distinct high-temperature response. The plateau indicates that, over 180–260 °C, the weld zone is not the strength-limiting region under this test configuration. The low value at T1 is consistent with insufficient fusion, as all three T1 specimens fractured in a brittle manner in the weld. Because each temperature is represented by a single joint (three specimens per joint), differences among neighboring plateau temperatures cannot be resolved statistically; the discussion therefore does not treat the small differences between 180, 210, 230, and 260 °C as meaningful.

### 5.4. Interpretation and Limitations

At the low-temperature end, the small T1 bead, qualitative SEM discontinuities, and brittle weld fracture are consistent with insufficient fusion. The increased complex spectral difference indicates an altered reflection response relative to T4. Local dielectric heterogeneity and limited molecular interdiffusion are plausible contributors, not measured results.

Near 210–230 °C, the presented welds show larger, more regular beads and ductile fracture outside the weld. NWBC_A_ reaches 0.142506 at T4. Both complex difference metrics equal zero at T4 because that specimen defines their reference, rather than because its microstructure or dielectric state has been independently proven optimal.

At 260–300 °C, the larger bead and increased complex spectral difference are associated with high-temperature processing. T6 retains a ductile heat-affected-region fracture, with a mean strength slightly lower than the 180–260 °C plateau (22.9 ± 1.0 MPa, n = 3). The present observations cannot distinguish geometric effects from changes in dielectric or crystalline properties, nor can they confirm thermal degradation.

Across the six temperatures, NWBC_A_ changes nonmonotonically and the two reference-relative complex metrics increase toward both ends. These descriptors can flag differences from the selected 230 °C specimen within this dataset. Their transferability to other pipes, batches, joints, probe lift-offs, and reference specimens remains to be established.

The proposed physical pathways—dielectric heterogeneity, crystallinity changes, chain orientation, and thermal degradation—remain hypotheses because these properties were not measured directly. PE is a low-loss dielectric with a permittivity of approximately 2.3 and a loss tangent of about 0.001 at microwave frequencies [26,27], so the measured reflection contrast can be sensitive to even modest local changes in permittivity and loss. High-temperature transitions in PE melts [28] and the influence of crystallinity and thermal degradation on PE structure [29] have also been documented. Nevertheless, direct dielectric spectroscopy and DSC/XRD characterization are required to verify the proposed origins of the observed spectral deviations; such measurements, together with replicate joints, are identified as priorities for future work.

## 6. Conclusions

(1) Wideband 6–14 GHz scanning and three derived metrics described temperature-dependent reflection differences in this PE100 DN200 specimen series. Background correction was implemented mathematically; a practical robustness benefit has not yet been demonstrated. In this single-factor series, welding temperature was the only intentionally varied parameter, although uncontrolled specimen-to-specimen variability may still contribute to the observed differences;

(2) Relative to the 230 °C specimen, CSD-RMS and BG-CSD-RMS increased toward both low and high temperatures. Their zero values at 230 °C follow from the definition of the reference metrics and are not an independent validation of joint quality;

(3) The low-temperature and high-temperature joints differed in bead appearance and tensile fracture mode. SEM images provide qualitative supporting observations, but dielectric changes, crystallinity, chain orientation, and degradation remain unmeasured proposed explanations;

(4) Bead size and NWBCA alone did not uniquely characterize joint performance across the tested temperatures. Geometry and local material response may both contribute to the microwave signatures;

(5) T1 fractured in a brittle manner in the weld, whereas T6 fractured in a ductile manner in the heat-affected region. Similar reference-relative spectral distances therefore do not imply the same failure mechanism or establish a stand-alone classifier;

(6) These results motivate further studies with replicate joints, independently characterized dielectric and microstructural properties, varied lift-off and pipe batches, and blinded performance testing before field screening or defect classification is claimed.

## Figures and Tables

**Table 1:** Welding processing parameters for the butt-fusion specimens.

Specimen	Welding Temperature (°C)	Pressure (MPa)	Heating Time (s)	Changeover Time (s)	Process Condition
T1	150	2.64	182	8	Severe low-temperature fusion deficiency
T2	180	2.64	182	8	Reduced low-temperature effect
T3	210	2.64	182	8	Near the lower limit of the nominal range
T4	230	2.64	182	8	Nominal reference
T5	260	2.64	182	8	Tendency toward over-welding
T6	300	2.64	182	8	Over-welding/HAZ damage risk

**Table 2:** Standardized regions used in microwave line-scan analysis.

Axial Range (mm)	Region	Description
0–30	Left parent material/background	Parent-material background used to suppress material and curvature effects
30–35	Left HAZ	Transition region modified by welding heat
35–45	Weld core W	Central fusion interface and target region
45–50	Right HAZ	Transition region modified by welding heat
50–80	Right parent material/background	Combined with the left side as background B to reduce unilateral bias

**Table 3:** Symbol explanation for microwave signal processing variables and evaluation indicators.

Symbol/ Indicator	Meanings	Processing Method or Physical Meaning
x, y	Spatial scanning coordinates	x represents the axial linear scanning position; y represents the circumferential position or the repetitive scanning position.
f_k_, k, K	Frequency points and total number of frequency points	f_k_ is the k frequency point, K = 201 in the 6–14 GHz range.
Re, Im	Real and imaginary parts of a complex signal	Complex reflection signal component output by the instrument
Z_i_(f_k_)	Single-point multiple microwave signal	Z = Re + jIm, amplitude and phase information
A_i_(f_k_), φ_i_(f_k_)	Single-point amplitude and phase	$A=\sqrt{Re^{2}+Im^{2}}$, φ = atan2(Im, Re)
W, B	Weld zone and base material background area	W = 35–45 mm, B = 0–30 mm merges with 50–80 mm
${\bar{A}}_{W},{\bar{A}}_{B}$	Regional average amplitude	The average amplitude of the weld zone and the base material background region at each frequency point
${\bar{Z}}_{W},{\bar{Z}}_{B}$	Regional average complex signal	The average complex-valued spectrum for both the weld zone and the base material background region at each frequency point
NWBC_A_	Normalized weld-to-background amplitude contrast	Characterizes the full-frequency-band amplitude difference between the weld zone and the base material background region.
CSD-RMS	Complex spectral difference root-mean-square value	Characterizes the complex spectral deviation of the weld zone of the test specimen relative to the T4 standard weld.
BG-CSD-RMS	Background-corrected complex spectral difference root-mean-square value	Subtracting the background signal from this test specimen’s base material and then comparing the result with that of the T4 standard specimen constitutes the core indicator for identifying localized anomalies in the weld seam in this study.

**Table 4:** Microwave evaluation metrics for all specimens.

Specimen	Temperature (°C)	NWBC_A_	CSD-RMS	BG-CSD-RMS
T1	150	0.037172	0.061735	0.057864
T2	180	0.086814	0.043042	0.036361
T3	210	0.097844	0.024298	0.024261
T4	230	0.142506	0.000000	0.000000
T5	260	0.126838	0.020289	0.023614
T6	300	0.093635	0.043885	0.048464

**Table 5:** Type-A tensile test results (mean ± SD; n = 3 per joint).

Specimen	Temperature (°C)	Maximum Force (kN)	Tensile Strength (MPa)	Fracture Location	Failure Mode	Assessment
T1	150	9.69 ± 0.19	20.4 ± 0.2	Weld	Brittle	Low-temperature cold-weld failure
T2	180	11.27 ± 0.06	23.2 ± 0.1	HAZ	Ductile	Reduced low-temperature effect
T3	210	11.13 ± 0.06	23.3 ± 0.1	HAZ	Ductile	Near nominal
T4	230	11.27 ± 0.21	23.4 ± 0.2	HAZ	Ductile	Nominal reference
T5	260	11.00 ± 0.52	23.4 ± 0.6	HAZ	Ductile	Tendency toward over-welding
T6	300	9.68 ± 0.01	22.9 ± 1.0	HAZ	Ductile	Over-welding/HAZ damage risk

## Data Availability

The data presented in this study are available from the corresponding author upon reasonable request.

## Abbreviations

The following abbreviations are used in this manuscript:

PE	Polyethylene
HDPE	High-density polyethylene
SDR	Standard dimension ratio
DN	Nominal diameter
SEM	Scanning electron microscopy
HAZ	Heat-affected zone
NWBCA	Normalized weld-to-background amplitude contrast
CSD-RMS	Complex spectral difference root mean square
BG-CSD-RMS	Background-corrected complex spectral difference root mean square
